# Measuring shame in eating disorders: confirmatory factor analyses and psychometric properties of a new internal and external shame scale

**DOI:** 10.1007/s40519-025-01759-8

**Published:** 2025-07-17

**Authors:** Olivia Keane, Emanuele Fino, Chérie Armour

**Affiliations:** 1 NTU Psychology, Nottingham Trent University, Nottingham, UK; 2 School of Psychology, Queen’s University Belfast, David Keir Building, 18-30 Malone Road, Belfast, BT9 5BN UK

**Keywords:** Shame, Eating disorders, Personality, Negative affect, Detachment

## Abstract

**Purpose:**

Feelings of shame can play a role in the development and maintenance of mental disorders. However, the role and measurement of shame in relation to eating disorders remain poorly understood. The current work presents the adaptation of a measure of external and internal shame in relation to eating disorders (EISS-ED) based on an integrative perspective that leverages the strengths of the biopsychosocial model and shame-pride cycle framework. Specifically, the EISS-ED accounts for how individuals believe they are perceived by others (external shame) and how they perceive themselves (internal shame) in relation to eating.

**Methods:**

The study used confirmatory factor analyses to examine the factor structure and psychometric properties of the EISS-ED (*N* = 478), Spearman’s correlations and Receiver Operator Characteristic curves were used for validity testing.

**Results:**

The results supported the two-factor structure (including 12 items) of the EISS-ED and its factorial invariance by clinical history and gender. Concurrent validity analyses showed positive correlations between external and internal shame and eating disorder symptoms, negative affect, and detachment. Both external and internal shame measures provided fair classification accuracy of participants based on self-reported history of clinical assessment or treatment for eating disorders.

**Conclusions:**

These findings improve the understanding of the role of shame in eating disorders and offer evidence on a valid assessment that can aid in identifying, measuring, and addressing this important construct, supporting targeted interventions.

*Level of evidence:* Level V, descriptive study.

## Introduction

Shame is a complex and multifaceted emotion that often stems from an individual’s sense of having failed to meet subjective and societal standards [1]. It is associated with feelings of inadequacy and worthlessness, and sometimes with self-harm. It has also been indicated as a risk factor for several mental conditions [2, 3]. Unlike guilt, which is typically linked to contingent feelings of regret, shame is pervasive and self-directed [1]. Shame has also been linked to eating disorders (EDs), specifically food control, to cope with negative emotions [4–6]. The current work presents a novel measure of shame in relation to EDs, based on an integrative approach that leverages the strengths of the biopsychosocial model and shame-pride cycle conceptual framework.

### Shame and eating disorders

Several ED symptoms concern emotions and behaviours associated with food consumption [7]. Individuals with EDs tend to report high levels of shame in relation to food consumption, a consequence of viewing themselves as a failure and lacking control [8–11]. In this regard, shame is intended to control eating and cope with external pressures or negative emotions, and its role in EDs can be better understood through established theoretical frameworks, such as the biopsychosocial model [12] and the shame-pride cycle [13]. The biopsychosocial model defines shame in relation to an individual’s need to belong, specifically, how they perceive themselves to appear to others [12]. Feelings of exclusion or neglect from others can lead to experiencing shame and behaving in ways aimed at reducing or avoiding shame. This model suggests that the primary function of shame is to prevent individuals from perceiving themselves as socially undesirable [12]. Such perceptions can trigger a maladaptive mechanism that favours food control through restriction and/or binging-purging behaviours, especially in vulnerable individuals like adolescents and young adults, for whom peer pressure, group norms and expectations facilitate the development of mental disorders [7]. On the other hand, the shame-pride cycle model postulates that individuals may associate feelings of shame with a specific behaviour when they perceive that behaviour as a negative reflection of themselves [13]. In the context of EDs, individuals may restrict food intake to alleviate feelings of shame associated with eating, considered as a form of poor self-control. Whilst food restrictions may temporarily decrease shame and increase pride, this maladaptive cycle can lead to the development of ED.

However, to date, these hypotheses have remained broadly speculative. Clarifying the role of shame in the psychopathology of EDs would provide greater insight for researchers and clinicians when deciding on treatment targets, allowing for personalised treatment. Clearly, this depends on whether reliable and valid measures of shame in relation to EDs are available, and to date, the literature on this topic remains limited. In particular, the biopsychosocial model and the shame-pride cycle offer different yet complementary conceptualisations of shame. Whilst the biopsychosocial model defines shame in terms of how individuals perceive that they appear to others (*external shame*), the shame-pride cycle considers shame as how an individual perceives themselves (*internal shame*). External and internal shame represent distinct but correlated sets of other- and self-perceptions [2, 14, 15]. A theoretical and measurement model of shame that leverages the strengths of the two perspectives could provide a comprehensive understanding of how shame operates in relation to EDs and pave the way for effective operationalisation of the construct.

The *External and Internal Shame Scale* (EISS [2]) represents a reliable and valid assessment that focuses on the distinction between these separate yet correlated dimensions of shame. The EISS integrates the conceptual strengths of both the biopsychosocial model and shame-pride cycle conceptual framework, with its brevity allowing for a parsimonious assessment of shame in clinical and community settings [2, 14]. Sixteen EISS items were initially developed to assess external and internal shame across four key domains, namely inferiority/inadequacy, exclusion, emptiness, and criticism, drawing on theoretical frameworks and clinical insights. Confirmatory factor analyses supported a reduced eight-item model that effectively captured both external and internal shame, exhibiting excellent psychometric properties. The sub-scales and overall score showed strong internal consistency, with Cronbach’s alpha values ranging from 0.82 to 0.89 and item-total correlations between 0.55 and 0.75. Evidence of construct validity was provided by positive correlations between the external shame sub-scale and the *Others As Shamer Scale-2* (OAS-2 [16]; *r* = 0.80) and between the internal shame sub-scale and the *Self-Criticizing & Self-Reassuring* scale (FSCRS [17]; *r* = 0.69). Additionally, both sub-scales were moderately correlated with depressive symptoms, as measured by the DASS-21 ([18]; in both cases, *r* = 0.57), supporting their concurrent validity. Moreover, the findings showed that women scored significantly higher than men on both external (*t*[663] =  −3.77, *p* < 0.001, *d* = 0.36) and internal shame (*t*[663] = −5.70, *p* < 0.001, *d* = 0.56).

However, while the EISS measures general feelings of shame, there is currently no equivalent tool specifically designed to measure shame in the context of EDs. While existing general measures of shame have been helpful in linking shame to EDs, they do not fully capture the context-specific nature of shame in individuals with EDs. Shame in EDs is often closely tied to highly specific triggers, such as food consumption, body image, eating habits, and perceived failures in self-control around eating [19, 20]. These are qualitatively distinct from more general interpersonal or trait-based experiences of shame assessed by traditional measures. Findings from a recent meta-analysis confirm these associations, suggesting that shame in EDs is not only pervasive but also context-specific, frequently occurring in relation to eating, body-related comparisons, or eating contexts [20]. For example, external shame in EDs may involve beliefs that others view one as weak, greedy, or lacking willpower due to eating habits, while internal shame may manifest as self-directed contempt and disgust specifically tied to eating behaviour and body image. Therefore, although general measures such as the EISS assess broad domains of shame, they do not directly capture triggers that are common in EDs, a gap that the current study sought to address.

### Eating disorders, shame, and maladaptive personality

Previous literature identified positive correlations between ED severity and personality traits, in particular, negative affect [21–23] and detachment [24, 25] from the DSM-5 model of maladaptive personality, measured through the *Personality Inventory for DSM-5* (PID-5 [26]). This is a dimensional model that includes five traits, namely: negative affect, detachment, psychoticism, antagonism, and disinhibition. Negative affect is defined as an individual’s tendency to experience a wide range of intense negative emotions, including anxiety and worry [27]. Earlier studies have shown a positive correlation between negative affect and both bulimia nervosa [28, 29] and anorexia nervosa [25, 29], confirmed across several studies among student populations [30–32]. Negative affect is associated with heightened maladaptive cognition, emotion, and behaviour involved in psychological disorders [33]. It is therefore reasonable to hypothesise that negative affect may play a role in the cycle of internal shame associated with disordered eating, especially in reinforcing the tendency to engage in maladaptive eating behaviours as a coping mechanism for feelings of inadequacy, low self-esteem, and emotional distress.

Detachment is characterised by withdrawal from social interactions and emotional experiences, a tendency for social avoidance, emotional coldness, and a lack of enjoyment of interpersonal relationships. Previous studies have found that detachment positively correlates with ED severity [24, 25]. This correlation may stem from detachment influencing how individuals perceive others, leading to feelings of social inadequacy. In turn, these feelings contribute to a selective numbing of bodily and emotional experiences as a coping mechanism, potentially linking detachment to external shame [34].

### Research objectives

The current work presents the adaptation of a measure of external and internal shame in relation to EDs (EISS-ED), based on an integrative theoretical model that leverages the strengths of both the biopsychosocial model and the shame-pride cycle conceptual framework. Specifically, the EISS-ED adapts the original EISS items to the context of eating, hypothesising a two-dimensional factor structure, which accounts  for perceptions of external and internal shame. In adapting the EISS to create the EISS-ED, the research aimed to retain the theoretically grounded structure of internal and external shame while enhancing content validity for an ED population. This was pursued by rephrasing the original EISS items to refer specifically to eating-related experiences and adding four new items derived from a review of ED literature and conceptual insights into how shame manifests in relation to food, eating behaviour, and body image.

Using two independent data collections, one with university students and one with a general population sample, the research employed a stratified random split method to randomly generate two sub-samples, with approximately equal sample size and representation in terms of clinical history (self-reported history of assessment or treatment for an ED vs. none), gender (female, male, other), and population (i.e., student vs. general). These were used to test the theoretical model via Confirmatory Factor Analysis (CFA) in Study 1 and to replicate the model in Study 2. In Study 3, the full combined sample was used to assess measurement invariance across clinical history, gender (limited to female vs. male due to sample size constraints), and population. Study 3 evaluated the validity of the EISS-ED by examining whether internal and external shame are positively associated with ED risk, as measured by two ED scales. Additionally, external shame was expected to correlate with detachment, and internal shame with negative affect. Based on prior research, moderate effect sizes were anticipated (ranging from 0.30 to 0.50 [5, 25, 29]). Finally, the postdictive validity of the EISS-ED was tested by examining its ability to distinguish individuals based on their past self-reported clinical history.

## Methods

### Participants and procedure

Participants were recruited through two independent data collections. The first data collection was with university students, via an institutional research participant scheme at a UK university, alongside social media advertisement, word of mouth, and ED online forums. Inclusion criteria were to be aged ≥ 18 years and currently enrolled as a student in a UK university. They were informed that this was a study on shame in relation to EDs, invited to participate on a voluntary basis and assured that declining participation would not result in any penalties. Students recruited via the institutional research participant scheme were compensated with two research credits for their final year project, whereas no credits or financial incentives were offered to the others. A total of 387 individuals expressed interest in participating,  of whom 284 met the inclusion criteria. Of these, 209 completed the full procedure, and their data were included in the final analysis. These were 166 (79.43%) individuals who self-identified as female, 32 (15.31%) male, and 11 (5.26%) either non-binary or other. Their age ranged between 18 and 35 years (*M*_age_ = 20.69; *SD*_*age*_ = 3.26). Moreover, 160 (76.56%) reported no history of clinical assessment or treatment for EDs vs. 49 who did (23.44%). The second data collection recruited participants from the general population via social media advertisement, word of mouth, and ED specialist online forums. Inclusion criteria were to be aged ≥ 18 years. Participation was voluntary and no incentives were offered. Initially, a total of 419 individuals expressed interested in participating.Of these, 269 completed the procedure. Regarding gender, 209 self-identified as female (77.70%), 34 male (12.64%) and 26 non-binary or other (9.67%). Their age ranged between 18 and 63 years (*M*_age_ = 26.35; *SD*_*age*_ = 8.30). Finally, 122 (45.35%) reported no history of clinical assessment or treatment for EDs, while 147 (54.65%) reported such a history.

Overall, the sample used in the analyses included 478 individuals who completed the procedure. A random split, stratified by clinical history, gender, and population, was used to generate two sub-samples (*N*_*1*_ = 241 and *N*_*2*_ = 237 participants). Table 1 presents demographic characteristics split by sub-sample.

**Table 1:** Demographic characteristics of participants

Sub-sample	*N*_*1*_ = 241	*N*_*2*_ = 237
Sample^a^		
General population	135 (56.02%)	134 (56.54%)
Student population	106 (43.98%)	103 (43.46%)
Gender^a^		
Female	189 (78.42%)	186 (78.48%)
Male	33 (13.69%)	33 (13.92%)
Other	19 (7.88%)	18 (7.59%)
Clinical History^a^		
No history	142 (58.92%)	140 (59.07%)
History	99 (41.08%)	97 (40.93%)
Age^b^	23.76 (7.15)	24.00 (7.17)

^a^n (%)

^b^Mean (SD)

The procedure lasted about 15 min and consisted of completing an online survey hosted through *Qualtrics.com*. Upon completion, participants were thanked, debriefed, and signposted to available mental health support services, in case they needed them.

### Materials and measures

All 16 original EISS items were adapted to specifically assess shame within the context of EDs for the EISS-ED. Additionally, two new items were developed to capture external shame, and two items to capture internal shame, informed by a literature review on the behavioural dimensions of shame in EDs [4, 13, 20], resulting in an initial pool of 20 items. Although only eight items were retained in the original EISS validation study, the full pool of 16 items was included in this adaptation to ensure broader content coverage in the early stages of scale adaptation and to allow for a comprehensive empirical evaluation of item performance in the ED context.

All items underwent peer review by the researchers and a group of undergraduate students. This procedure aimed to ensure clarity of wording, address potential redundancy, and maximise understanding. The revised version was piloted among five students, who were invited to read and evaluate the quality of each item and to report any concerns regarding the intended meaning or format of the items. The process resulted in a 20-item version for testing. Ten items measured external shame (e.g., *Other people judge the amount of food I eat*), while the remaining ten items measured internal shame (e.g., *I must justify my appetite*). The EISS-ED uses a 5-point Likert scale (0 = *Never*, 4 = *Always*) to indicate how frequently participants experience each described feeling. EISS-ED scores are calculated by summing up participants’ responses to either total scale scores or sub-scale scores.

The first data collection (student sample) included the SCOFF [35] to measure ED risk (McDonald’s omega = 0.72 [0.66–0.78]). This is a 5-item scale (e.g*., Would you say that food dominates your life?*), designed to screen for ED risk within the general population. It uses a binary response format (0 = *No,* 1 = *Yes*). Total scores are obtained by summing individual items’ scores.

The second data collection (general population) included the *Eating Disorder Examination Questionnaire* (EDE-Q [36]). This is a 28-item measure of the range, frequency, and severity of ED symptoms. Twenty-three items (e.g., *In the past 28 days, how often have you had a definite desire to have a totally flat stomach?*) are rated on a 7-point scale (1 = *no days*, 7 = *every day*), underlying four sub-scales, measuring restraint (omega = 0.90 [0.87–0.92]), eating concern (omega = 0.86 [0.83–0.89]), shape concern (omega = 0.94 [0.92–0.95]) and weight concern (omega = 0.85 [0.82–0.89]). Due to a technical error, one of the items (Item 8) was not included. Sub-scale scores were calculated by averaging item scores.

The present study used a 24-item selection from the full *Personality Inventory for DSM-5* (PID-5 [26]) to assess two maladaptive personality traits: negative affect (omega across student and general samples: 0.89 [0.88–0.91]) and detachment (omega across student and general samples: 0.86 [0.83–0.89]). Each domain is assessed using 12 items (e.g., *I don’t like to get too close to people*), drawn from three core facets per domain (i.e., emotional lability, anxiousness, and separation insecurity for negative affect; withdrawal, anhedonia, and intimacy avoidance for detachment). Items were rated on a 5-point scale (0 = *Very False* to 4 = *Very True*). The present study only used the negative affect and detachment sub-scale to test targeted hypotheses. Finally, participants were asked to self-report whether they have ever been assessed or received a clinical assessment or diagnosis of an ED  or related symptoms (0 = *No,* 1 = *Yes*).

### Analytical plan

Studies 1 and 2 used Confirmatory Factor Analysis (CFA) with weighted least squares estimation and mean and variance adjustment (WLSMV; theta parameterisation), appropriate for ordinal data. To evaluate the goodness of fit of the model, the following indices (cut-off values) were used [37]: Comparative Fit Index (CFI ≥ 0.95), Tucker-Lewis Fit Index (TLI ≥ 0.95), Root Mean Square Error of Approximation with 90% confidence intervals (RMSEA, with upper bound < 0.08), and Standardised Root Mean Squared Residual (SRMR < 0.08), in their robust versions [38]. Internal consistency of all measures was evaluated through omega values, including 95% confidence intervals (MLR method). Study 3 used Spearman’s method for correlations and Receiver Operating Characteristic (ROC) curves for postdictive validity analyses. Measurement invariance was assessed by fitting, evaluating, and comparing a series of increasingly constrained models: configural (no constraints across groups), metric (constraining thresholds and factor loadings), and scalar (constraining thresholds, loadings, and intercepts). A change of ≥ 0.010 in the scaled CFI (DCFI), together with a change of ≥ 0.015 in the scaled RMSEA (DRMSEA) were considered as indicative of non-invariance [39].

All analyses were run in *R* version 4.3.1 [40] and *RStudio* version 2024.04.2 [41], including the *lavaan* [42]*, semTools* [43]*,* and *pROC* packages [44].

## Study 1—Results

Table 2 presents demographic characteristics split by sub-sample.

**Table 2:** Descriptive statistics and intercorrelations

Item	Sub-sample 1 (*N* = 241)				Sub-sample 2 (*N* = 237)				1	2	3	4	5	6
	*M*	*SD*	Skewness	Kurtosis	*M*	*SD*	Skewness	Kurtosis						
1. I feel inadequate to others after I have eaten	2.20	1.41	0.78	−0.85	2.43	1.51	0.57	−1.20		0.73***	0.59***	0.24***	0.74***	0.58***
2. I feel flawed as a person when looking back on my food-consumption	3.00	1.54	-0.01	−1.53	3.18	1.54	-0.19	−1.50	0.69***		0.65***	0.30***	0.72***	0.64***
3. I feel like I must justify my appetite^a^	2.92	1.52	0.10	−1.49	3.11	1.53	-0.11	−1.48	0.63***	0.64***		0.27***	0.61***	0.49***
4. I feel that I eat a lot more than others^b^	2.35	1.19	0.72	−0.40	2.38	1.23	0.67	−0.61	0.20**	0.29***	0.10		0.26***	0.29***
5. I feel unworthy as a person after I have eaten	2.23	1.39	0.83	−0.72	2.55	1.56	0.47	−1.36	0.75***	0.72***	0.64***	0.25***		0.65***
6. I feel empty inside when looking back at what I have eaten	2.29	1.37	0.73	−0.77	2.56	1.48	0.41	−1.31	0.66***	0.74***	0.60***	0.22***	0.71***	
7. I am judgemental of myself after I have eaten	3.17	1.47	-0.06	−1.46	3.56	1.46	-0.51	−1.24	0.70***	0.80***	0.65***	0.32***	0.77***	0.72***
8. I criticise myself when thinking about my eating habits	3.45	1.40	-0.32	−1.34	3.67	1.44	-0.59	−1.15	0.64***	0.82***	0.63***	0.30***	0.70***	0.70***
9. My insecurities are based on what I eat^a^	2.68	1.31	0.27	−1.15	2.83	1.42	0.18	−1.30	0.61***	0.70***	0.56***	0.24***	0.69***	0.69***
10. I need to hide myself after I have eaten	2.16	1.34	0.91	−0.46	2.47	1.51	0.54	−1.22	0.66***	0.67***	0.58***	0.30***	0.75***	0.68***
11. Other people see me as inferior to them because of my appetite	1.79	1.22	1.49	1.00	1.82	1.18	1.39	0.89	0.52***	0.48***	0.40***	0.21**	0.52***	0.50***
12. Other people see my food choices as not being up to their standards	2.28	1.31	0.75	−0.66	2.48	1.37	0.50	−1.07	0.46***	0.40***	0.33***	0.05	0.41***	0.47***
13. Others think that my food choices greatly differ from theirs^b^	2.87	1.32	0.16	−1.21	2.84	1.38	0.17	−1.30	0.42***	0.45***	0.42***	0.02	0.43***	0.48***
14. Others don’t understand the food choices I make for myself	2.79	1.37	0.28	−1.24	2.84	1.42	0.14	−1.36	0.46***	0.58***	0.45***	0.08	0.55***	0.55***
15. Other people see me as undeserving because of my food choices	1.58	0.98	1.77	2.28	1.68	1.10	1.64	1.67	0.53***	0.40***	0.38***	0.18**	0.51***	0.45***
16. Other people think that I am useless when they see what I eat	1.58	0.98	1.67	1.84	1.78	1.22	1.36	0.55	0.59***	0.47***	0.41***	0.16*	0.58***	0.49***
17. Other people judge the amount of food I eat	2.65	1.37	0.43	−1.10	2.70	1.38	0.36	−1.16	0.58***	0.57***	0.54***	0.24***	0.57***	0.56***
18. Other people are disappointed in me when they see me eat	1.83	1.18	1.38	0.84	1.83	1.18	1.30	0.51	0.51***	0.48***	0.39***	0.20**	0.50***	0.49***
19. Other people are critical about my eating habits	2.49	1.27	0.61	−0.68	2.65	1.34	0.30	−1.23	0.50***	0.50***	0.45***	0.06	0.51***	0.53***
20. Other people are embarrassed to be eating with me	1.52	0.98	2.06	3.48	1.58	1.01	1.90	2.90	0.42***	0.39***	0.31***	0.18**	0.44***	0.38***

Item	7	8	9	10	11	12	13	14	15	16	17	18	19	20
1. I feel inadequate to others after I have eaten	0.70***	0.64***	0.69***	0.61***	0.51***	0.41***	0.34***	0.46***	0.44***	0.48***	0.52***	0.46***	0.44***	0.43***
2. I feel flawed as a person when looking back on my food-consumption	0.78***	0.78***	0.72***	0.63***	0.52***	0.54***	0.47***	0.63***	0.44***	0.45***	0.63***	0.55***	0.57***	0.46***
3. I feel like I must justify my appetite^a^	0.68***	0.66***	0.62***	0.58***	0.45***	0.45***	0.43***	0.52***	0.38***	0.43***	0.53***	0.41***	0.47***	0.38***
4. I feel that I eat a lot more than others^b^	0.29***	0.30***	0.29***	0.25***	0.30***	0.12	0.13*	0.19**	0.29***	0.24***	0.21**	0.26***	0.12	0.26***
5. I feel unworthy as a person after I have eaten	0.72***	0.67***	0.66***	0.69***	0.48***	0.38***	0.35***	0.48***	0.43***	0.46***	0.48***	0.45***	0.42***	0.44***
6. I feel empty inside when looking back at what I have eaten	0.61***	0.62***	0.63***	0.52***	0.48***	0.36***	0.33***	0.44***	0.43***	0.41***	0.45***	0.44***	0.38***	0.32***
7. I am judgemental of myself after I have eaten		0.81***	0.76***	0.63***	0.44***	0.42***	0.40***	0.53***	0.42***	0.44***	0.54***	0.46***	0.49***	0.38***
8. I criticise myself when thinking about my eating habits	0.84***		0.70***	0.59***	0.44***	0.43***	0.44***	0.59***	0.41***	0.46***	0.57***	0.45***	0.52***	0.40***
9. My insecurities are based on what I eat^a^	0.78***	0.72***		0.61***	0.41***	0.39***	0.39***	0.50***	0.40***	0.42***	0.50***	0.45***	0.49***	0.35***
10. I need to hide myself after I have eaten	0.77***	0.69***	0.64***		0.47***	0.39***	0.38***	0.50***	0.47***	0.42***	0.48***	0.45***	0.47***	0.43***
11. Other people see me as inferior to them because of my appetite	0.48***	0.50***	0.50***	0.46***		0.50***	0.41***	0.49***	0.55***	0.58***	0.53***	0.60***	0.50***	0.49***
12. Other people see my food choices as not being up to their standards	0.36***	0.41***	0.36***	0.38***	0.55***		0.60***	0.63***	0.43***	0.48***	0.58***	0.51***	0.62***	0.50***
13. Others think that my food choices greatly differ from theirs^b^	0.42***	0.49***	0.43***	0.42***	0.46***	0.62***		0.66***	0.34***	0.36***	0.52***	0.40***	0.55***	0.37***
14. Others don’t understand the food choices I make for myself	0.57***	0.59***	0.52***	0.52***	0.48***	0.57***	0.71***		0.35***	0.39***	0.62***	0.47***	0.67***	0.39***
15. Other people see me as undeserving because of my food choices	0.37***	0.40***	0.36***	0.38***	0.73***	0.57***	0.44***	0.40***		0.63***	0.54***	0.52***	0.43***	0.57***
16. Other people think that I am useless when they see what I eat	0.43***	0.43***	0.44***	0.44***	0.74***	0.59***	0.49***	0.47***	0.75***		0.49***	0.57***	0.38***	0.50***
17. Other people judge the amount of food I eat	0.60***	0.60***	0.51***	0.55***	0.59***	0.64***	0.55***	0.58***	0.61***	0.60***		0.53***	0.64***	0.48***
18. Other people are disappointed in me when they see me eat	0.43***	0.43***	0.47***	0.45***	0.59***	0.57***	0.45***	0.43***	0.65***	0.65***	0.57***		0.50***	0.54***
19. Other people are critical about my eating habits	0.51***	0.55***	0.48***	0.48***	0.50***	0.66***	0.59***	0.57***	0.53***	0.54***	0.71***	0.52***		0.39***
20. Other people are embarrassed to be eating with me	0.33***	0.38***	0.36***	0.37***	0.54***	0.47***	0.46***	0.39***	0.67***	0.64***	0.54***	0.65***	0.53***	

^a^New items measuring *internal* shame in relation to eating disorders, not included in the original version of the EISS;

^b^New items measuring *external* shame in relation to eating disorders, not included in the original version of the EISS

This table presents Spearman correlation coefficients with pairwise deletion. The data were split by sub-sample (upper triangle = 1, *N* = 241; lower triangle = 2, *N* = 237). *p < 0.05, **p < 0.01, ***p < 0.001

The results of the CFA conducted in the first randomly selected sub-sample (*N*_*1*_ = 241) indicated unsatisfactory fit of the measurement model (*Chi-square* = 315.28, *df* = 169, *p* < 0.001, CFI = 0.94, TLI = 0.93, RMSEA = 0.09 [0.07–0.10], SRMR = 0.05). To address this, the study undertook a series of adjustments to the original version of the EISS-ED, prioritising items without residual correlations and iteratively re-estimating model fit. The final model included 12 items: six measuring external shame and six measuring internal shame. The final version of the EISS-ED was obtained after removing one of the newly generated items (Item 4: *I feel that I eat a lot more than others*), and seven adapted items, including statements such as *I feel inadequate to others after I have eaten* (Item 1), *I feel unworthy as a person after I have eaten* (Item 5), *I am judgemental of myself after I have eaten* (Item 7), *Other people see my food choices as not being up to their standards* (Item 12), *Others don’t understand the food choices I make for myself* (Item 14), *Other people see me as undeserving because of my food choices* (Item 15), and *Other people are critical about my eating habits* (Item 19). This version showed excellent fit (Chi-square = 62.03, *df* = 53, *p* = 0.19, CFI = 0.99, TLI = 0.99, RMSEA = 0.03 [0.00–0.07], SRMR = 0.03). Each factor showed satisfactory omega reliability (external shame = 0.85 [0.82–0.89]; internal shame = 0.91 [0.89–0.93]) and the solution was highly interpretable.

## Study 2—Results

**Fig. 1:**
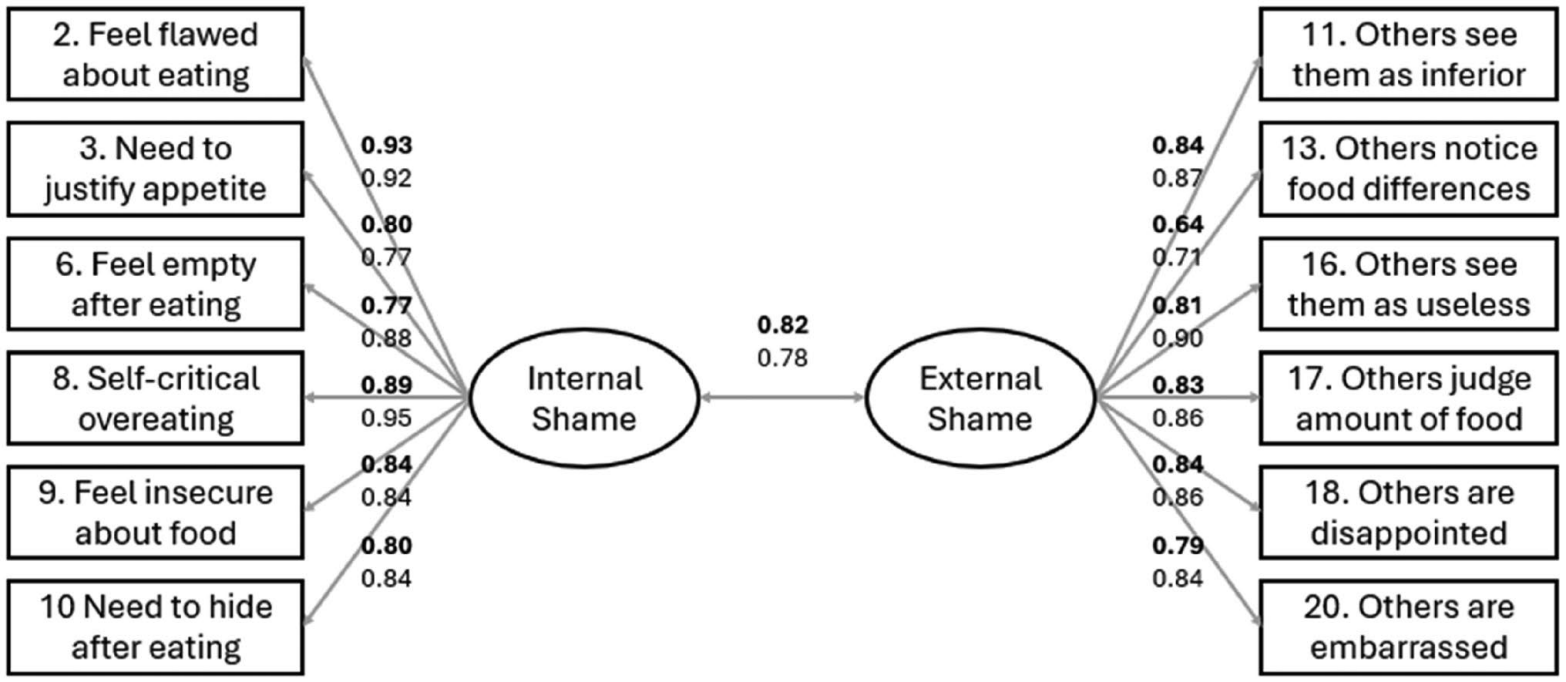
Confirmatory Factor Analysis (WLSMV estimation). Bolded values represent data from the first sub-sample (N = 241), while non-bolded values are from the second sub-sample (N = 237)

Study 2 replicated the model tested in Study 1, this time in the second randomly selected sub-sample (*N*_*2*_ = 237). The CFA confirmed the satisfactory fit of the retained model (Chi-square = 71.81, *df* = 53, *p* = 0.04, CFI = 1.00, TLI = 1.00, RMSEA = 0.02 [0.00–0.07], SRMR = 0.03). Figure 1 presents the measurement model and shows the factor loadings for both studies.

## Study 3

Study 3 tested measurement invariance (by gender, clinical history, and sample) and concurrent and postdictive validity of the EISS-ED. Invariance testing (*N* = 478) showed that the solution was invariant across groups defined by either previous clinical history (metric: DCFI = −0.001, DRMSEA = -0.001; scalar: DCFI = −0.001, DRMSEA = 0.000), gender (metric: DCFI = 0.000, DRMSEA = −0.010; scalar: DCFI = 0.000, DRMSEA = 0.000), or sample (metric: DCFI = 0.000, DRMSEA = −0.005; scalar: DCFI = −0.001, DRMSEA = 0.003; Table 3).

**Table 3:** Invariance testing (*N* = 478)

Grouping variable	Model	*Chi-square*	*df*	*p*	CFI	RMSEA	SRMR	DCFI	DRMSEA	DSRMR
Clinical history	Configural	146.17	106	0.006	1.00	0.04	0.04			
	Metric	191.74	140	0.002	1.00	0.04	0.04	−0.001	−0.001	0.001
	Scalar	204.59	150	0.002	0.99	0.04	0.04	−0.001	0.000	0.001
Gender	Configural	142.68	106	0.01	1.00	0.04	0.04			
	Metric	165.94	139	0.059	1.00	0.03	0.04	0.000	−0.010	0.000
	Scalar	179.38	149	0.045	1.00	0.03	0.04	0.000	0.000	0.000
Population	Configural	152.52	106	0.002	1.00	0.04	0.04			
	Metric	189.02	140	0.004	1.00	0.04	0.04	0.000	−0.005	0.001
	Scalar	209.15	150	0.001	0.99	0.04	0.04	−0.001	0.003	0.000

Correlation analysis showed positive intercorrelations between the two shame sub-scales’ scores (*r*_*s*_ = 0.72, *p* < 0.001, *N* = 478). Similarly, correlations were found between external shame and ED risk (measured by the SCOFF; *r*_*s*_ = 0.47, *p* < 0.001, *N* = 209) and ED symptomatology (measured by the EDE-Q; restraint: *r*_*s*_ = 0.32, *p* < 0.001; eating concerns *r*_*s*_ = 0.44, *p* < 0.001; weight concerns: *r*_*s*_ = 0.30, *p* < 0.001; shape concerns: *r*_*s*_ = 0.36, *p* < 0.001; *N* = 269) were positive but lower than the correlations observed between internal shame and the SCOFF (*r*_*s*_ = 0.71, *p* < 0.001, *N* = 209) and between internal shame and EDE-Q sub-components (restraint: *r*_*s*_ = 0.55, *p* < 0.001; eating concerns *r*_*s*_ = 0.69, *p* < 0.001; weight concerns: *r*_*s*_ = 0.50, *p* < 0.001; shape concerns: *r*_*s*_ = 0.57, *p* < 0.001; *N* = 269). Lower correlations were found between external shame and negative affect (*r*_*s*_ = 0.14, *p* = 0.002; *N* = 478) and between external shame and detachment (*r*_*s*_ = 0.27, *p* < 0.001; *N* = 478) compared to the correlations between internal shame and the two personality traits (respectively: *r*_*s*_ = 0.30, *p* < 0.001; *r*_*s*_ = 0.33, *p* < 0.001; *N* = 478). Table 4 presents detailed results from the correlation analyses.

**Table 4:** Concurrent validity analysis (*N* = 478)

Variable	1	2	3	4	5	6	7	8
1. External shame								
2. Internal shame	0.72***							
3. Negative affect	0.14**	0.30***						
4. Detachment	0.27***	0.33***	0.38***					
5. SCOFF	0.47***	0.71***	0.32***	0.34***				
6. EDE-Q restraint	0.32***	0.55***	0.36***	0.41***				
7. EDE-Q eating concern	0.44***	0.69***	0.56***	0.50***		0.72***		
8. EDE-Q weight concern	0.30***	0.50***	0.53***	0.45***		0.64***	0.75***	
9. EDE-Q shape concern	0.36***	0.57***	0.52***	0.51***		0.71***	0.81***	0.88***

This table presents Spearman correlation coefficients with pairwise deletion; all results involving SCOFF scores pertain to the student sample (*N* = 209), those involving EDE-Q scores to the general population sample (*N* = 269); *p < 0.05, **p < 0.01, ***p < 0.001

**Fig. 2:**
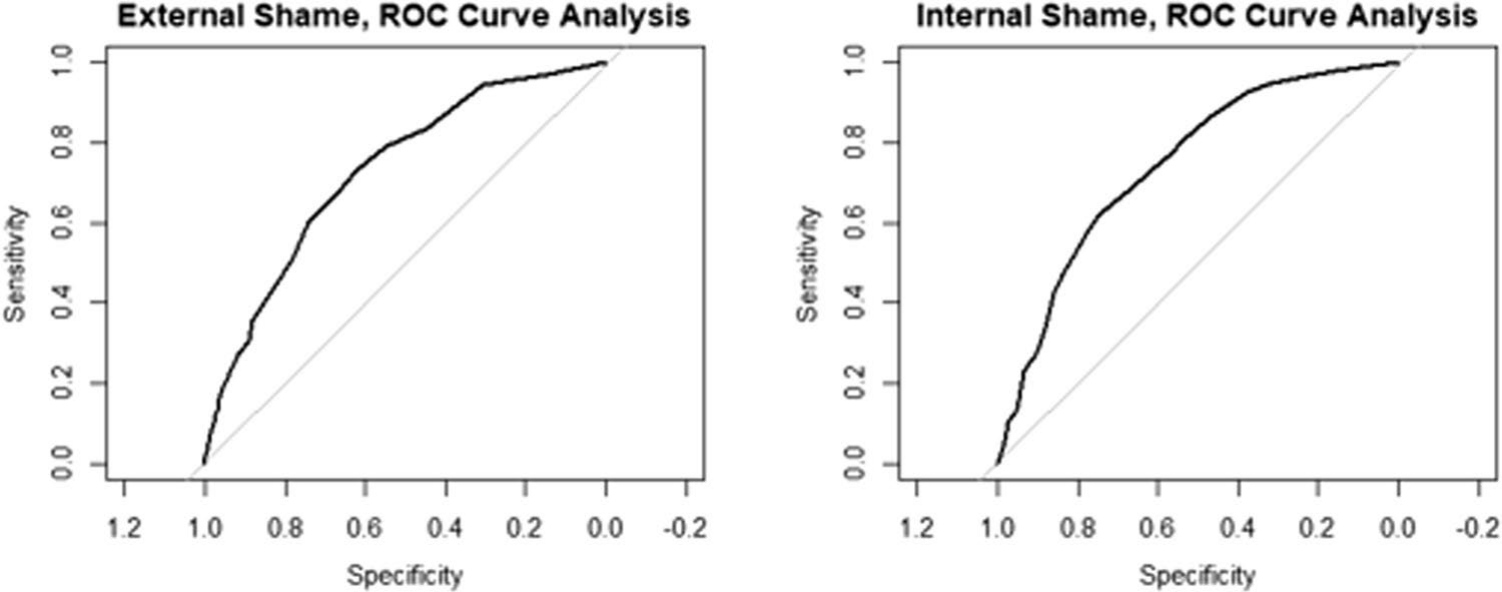
Receiving Operator Characteristic curve analysis (N = 478)

Generalised linear modelling and ROC analyses showed that both external shame (*b* = 0.15, *SE* = 0.02, *p* < 0.001, AUC = 0.73) and internal shame (*b* = 0.13, *SE* = 0.02, *p* < 0.001, AUC = 0.74) significantly predict clinical history and provide fairly accurate postdictive classification (Fig. 2).

## General discussion

The current work presented the adaptation of a measure of external and internal shame in relation to eating disorders (EISS-ED), based on an integrative theoretical perspective that leverages the strengths of the biopsychosocial model and the shame-pride cycle framework. The EISS-ED originally included 20 items designed to measure how individuals believe they are perceived by others (external shame) and how they perceive themselves (internal shame) in relation to controlling eating to cope with external pressures or negative emotions. Findings from confirmatory analyses supported the hypothesised two-factor structure across two randomly selected samples. The final model included 12 items: six measuring external shame and six measuring internal shame, demonstrating adequate construct and content validity. Each sub-scale showed adequate internal consistency, metric and scalar invariance across self-reported previous clinical history (i.e., having or not having a history of clinical assessment or treatment for EDs), gender (females and males), and population (student and general populations). Positive correlations were found between EISS-ED scores and ED risk (measured by the SCOFF [35]) and EDE-Q symptoms [36], including restraint, eating concerns, weight concerns, and shape concerns, as well as maladaptive personality traits [26] such as negative affect and detachment. In both domains, internal shame showed stronger correlations than external shame. Finally, evidence of postdictive validity indicated that both EISS-ED sub-scale scores accurately classify individuals based on previous clinical history.

The current work fills a critical gap in research and assessment of great importance for student populations, where there is a high prevalence of EDs [45–47]. The two-factor structure of the EISS-ED supports the proposed integrative theoretical perspective, which leverages the strengths of both the biopsychosocial model and the shame-pride cycle framework. This is in line with the assumptions of the original EISS [2], but adapted to measure shame specifically in relation to controlling eating as a coping strategy for external pressures or negative emotions. Consistently, these findings highlight the multidimensional nature of the construct and the need for multifactorial measurement models to account for these important yet distinct sub-dimensions. The findings from the current study also provide solid evidence of the concurrent validity of the EISS-ED, confirming the initial hypotheses. In fact, results from correlation analyses showed high correlations between the two EISS-ED sub-scales and that both sub-scales were significantly and positively correlated with measures of ED, negative affect, and detachment. This is consistent with previous literature that reported that individuals with ED have significantly higher levels of neuroticism [25, 29] and detachment [24, 25]. These correlations are also consistent with the well-known impact of maladaptive personality traits on individuals’ mental health and wellbeing, whereby higher levels of detachment are significantly and negatively associated with positive coping [48].

Internal shame correlated more strongly than external shame with all measures of EDs, neuroticism, and detachment. Similar findings were reported in the psychometric evaluation of the original EISS [2], whereby internal shame correlated higher than external shame with measures of anxiety and depression [2, 14]. Therefore, the link between internal and external shame and EDs may be conditional on the specific type of ED. In this regard, a study reported that individuals with anorexia nervosa had significantly greater levels of internal shame than external shame. Conversely, the pattern seemed inverted in those with bulimia nervosa [5]. Nevertheless, the influence of exposure to adverse childhood experiences upon the development of internal vs. external shame cannot be ruled out, and internal shame has been considered a key element of a treatment-resistant ED ecophenotype, characterised by higher severity and psychiatric comorbidity [49]. However, to avoid speculation, it is recommended that future research should replicate these findings across different ED types, aiming to shed light on the complex interplay between internal and external shame and ED manifestations.

The findings on the postdictive validity of the EISS-ED also support evidence from previous literature, i.e., that individuals with a clinical diagnosis of an ED tend to report greater levels of shame than those without a diagnosis [8, 9]. Considering the high prevalence of EDs in university students, these results are especially meaningful, as a reliable and valid tool for assessing shame, a key ED symptom, such as the EISS-ED, has the potential to inform future improvements in ED prevention, intervention, and policy where they are most needed. The EISS-ED also brings the advantage of using items that do not insist upon traditional ED symptomatology, such as weight loss or control and food restriction. This is of great importance and highly practical, considering that individuals with EDs may be more likely to experience distress when undertaking ED assessments, due to the sensitive nature of the questions being asked [50].

### Strength and limitations

The study has some limitations. First, the current study used relatively small samples of students and individuals from the general population. For this reason, the findings should not be generalised to clinical settings, at least not until further evidence confirms the validity of the EISS-ED among individuals with a confirmed ED diagnosis. Second, differences in recruitment of female and male participants require further consideration, especially given the known discrepancies in how EDs manifests in female versus male individuals [51, 52]. Third, correlations between the EISS-ED and objective measures of ED-related variables were not tested; accordingly, future research would benefit from considering its associations with body mass, body composition, or metabolic and nutritional markers. Fourth, we acknowledge that including the original EISS in future studies for direct comparison would allow for a valuable test of incremental validity, which should be a priority for future research, with the aim of determining whether the EISS-ED provides unique explanatory power in predicting disordered eating behaviours beyond the original EISS. Lastly, although the SCOFF and the EDE-Q represent established measures of risk for ED and ED-related symptoms, the results of validity analyses would require replication with broader and more comprehensive measures of ED symptoms and comorbid psychiatric conditions.

## Conclusions

The current study evidenced the reliability and validity of the EISS-ED in student and general population samples, highlighting its potential to significantly enhance the assessment of external and internal shame in the context of EDs. Drawing upon a solid theoretical basis that integrates the shame-pride cycle framework and the biopsychosocial model, the EISS-ED represents a valuable addition to the assessment literature on EDs. Further research is needed to replicate these findings in clinical settings and across various ED subtypes.

### What is already known on this subject?

Shame is a key but underexplored factor in EDs, influencing the development and maintenance of symptoms. Two dimensions of shame respectively relate to how individuals believe others view them (external shame), and how they view themselves as inadequate or having failed (internal shame). Theoretical models like the biopsychosocial model and the shame-pride cycle provide complementary frameworks for understanding the role of shame in EDs. While tools exist to measure shame as a general construct, a reliable, targeted assessment of internal and external shame specifically within the context of ED has been lacking, limiting understanding of its role in EDs and reducing opportunities for targeted intervention.

### What this study adds?

The External and Internal Shame Scale for Eating Disorders (EISS-ED) introduces a novel, evidence-based psychometric tool to assess shame, a key factor in eating disorders. By integrating the biopsychosocial model and shame-pride cycle framework, the EISS-ED enhances understanding of internal and external shame, supports reliable assessment and targeted intervention, and addresses a critical gap in ED research, overall supporting better outcomes for those affected.

## Data Availability

The datasets and R code generated and used in the current study are available in the Open Science Framework repository, at: https://osf.io/paqhn/?view_only = 4dd82cacf1434f9a87160cc1421f1555.
